# Distinct Gene Expression Signatures in Lynch Syndrome and Familial Colorectal Cancer Type X

**DOI:** 10.1371/journal.pone.0071755

**Published:** 2013-08-12

**Authors:** Mev Dominguez-Valentin, Christina Therkildsen, Srinivas Veerla, Mats Jönsson, Inge Bernstein, Åke Borg, Mef Nilbert

**Affiliations:** 1 Department of Oncology, Institute of Clinical Sciences, Lund University, Lund, Sweden; 2 The Danish HNPCC Register, Clinical Research Centre and Department of Gastroenterology, Copenhagen University Hospital, Hvidovre, Denmark; Faculty of Medicine, University of Porto, Portugal

## Abstract

**Introduction:**

Heredity is estimated to cause at least 20% of colorectal cancer. The hereditary nonpolyposis colorectal cancer subset is divided into Lynch syndrome and familial colorectal cancer type X (FCCTX) based on presence of mismatch repair (MMR) gene defects.

**Purpose:**

We addressed the gene expression signatures in colorectal cancer linked to Lynch syndrome and FCCTX with the aim to identify candidate genes and to map signaling pathways relevant in hereditary colorectal carcinogenesis.

**Experimental design:**

The 18 k whole-genome c-DNA-mediated annealing, selection, extension, and ligation (WG-DASL) assay was applied to 123 colorectal cancers, including 39 Lynch syndrome tumors and 37 FCCTX tumors. Target genes were technically validated using real-time quantitative RT-PCR (qRT-PCR) and the expression signature was validated in independent datasets.

**Results:**

Colorectal cancers linked to Lynch syndrome and FCCTX showed distinct gene expression profiles, which by significance analysis of microarrays (SAM) differed by 2188 genes. Functional pathways involved were related to G-protein coupled receptor signaling, oxidative phosphorylation, and cell cycle function and mitosis. qRT-PCR verified altered expression of the selected genes *NDUFA9, AXIN2, MYC, DNA2* and *H2AFZ*. Application of the 2188-gene signature to independent datasets showed strong correlation to MMR status.

**Conclusion:**

Distinct genetic profiles and deregulation of different canonical pathways apply to Lynch syndrome and FCCTX and key targets herein may be relevant to pursue for refined diagnostic and therapeutic strategies in hereditary colorectal cancer.

## Introduction

Heredity is a major risk factor for colorectal cancer. Identification of individuals and families at increased risk allows for targeted surveillance, which has been shown to reduce morbidity and mortality from colorectal cancer [1], [2]. Hereditary nonpolyposis colorectal cancer (HNPCC) accounts for 3–6% of colorectal cancer. The syndrome is clinically classified according to the Amsterdam criteria, which require at least three HNPCC-associated cancers, two affected first-degree relatives and at least one individual diagnosed before age 50 [3], [4]. Between one-third and half of the families that fulfill the Amsterdam criteria carry germline mismatch repair (MMR) gene mutations in *MLH1, MSH2, MSH6* or *PMS2* and are referred to as having Lynch syndrome [5]–[7]. The remaining families with a yet unidentified genetic background show a predominance of colorectal cancer, frequent synchronous and metachronous adenomatous polyps and few extracolonic tumors and are referred to as familial colorectal cancer type X (FCCTX) [8], [9]. Compared to Lynch syndrome, colorectal cancers linked to FCCTX develop later, predominantly occur in the distal colon and less often show the distinctive morphological features tumor-infiltrating lymphocytes, poor differentiation and mucin production characteristic of Lynch syndrome tumors [8], [10], [11].

Data on the genetic features and tumorigenic pathways of FCCTX are scarce though genomic studies have demonstrated mean 6–8 copy number alterations with recurrent gains of 7p, 7q, 8q, 13q, 20p and 20q and losses of 17p, 18p and 18q [9], [12], [13]. Two studies have addressed gene expression profiles and mutation patterns in FCCTX tumors and have described similarities between FCCTX and sporadic MMR stable tumors [14], [15]. In sporadic colorectal cancer, MMR defective tumors show distinct gene expression profiles with up-regulation of immunomodulatory genes, such as chaperones, cytokines and cytotoxic mediators, heat shock genes, major histocompatibility complex genes and apoptosis-related genes [16]–[18]. We applied global gene expression profiling in order to delineate candidate genes and differential involvement of pathways within the HNPCC subset of hereditary colorectal cancer with comparison between colorectal cancer linked to Lynch syndrome and FCCTX.

## Materials and Methods

### Ethics Statement

The project was ethically reviewed in Copenhagen, where a waiver for collection of tissues and clinical data was granted. All patients provided an informed consent for inclusion into the Danish HNPCC register during genetic counselling sessions. Ethical approval for the study was granted from the Scientific and Ethical Committee at The Capital Region of Copenhagen, Denmark (H-D-2007–0032).

### Sample Selection and RNA Extraction

The national Danish HNPCC register was used to identify colorectal cancers from individuals with Lynch syndrome and FCCTX. Lynch syndrome was defined as families/individuals with disease-predisposing germline MMR gene mutations (n = 16 in *MLH1*, n = 13 in *MSH2* and n = 10 in *MSH6*). Immunohistochemical MMR protein staining and microsatellite instability (MSI) analysis were performed as previously described [11], [13]. FCCTX tumors were defined as tumors that developed in families fulfilling the Amsterdam criteria, but showed no disease-predisposing MMR gene mutations and had retained MMR function. Sporadic colorectal cancers were selected to represent MMR deficient and MMR proficient tumors from individuals without family history of cancer. Clinical data are summarized in Table 1. Compared to Lynch syndrome tumors, FCCTX tumors were more often located in the left side of the colon (p<0.00001, Fisheŕs exact test) and showed high or moderate differentiation (p<0.00001, Fisheŕs exact test). Lynch syndrome and FCCTX did not differ as regards age at onset (mean 53 years versus 58 years) or tumor stage.

**Table 1:** Clinical characteristics of the colorectal cancer subsets.

Characteristics	Lynch syndrome	FCCTX	Sporadic MMR deficient	Sporadic MMR proficient
Number of tumors profiled	39	37	26	21
Mean (range) age at onset	53 (25–86)	58 (33–88)	74 (62–86)	69 (51–83)
Sex (% female)	54	38	54	57
Tumor location, proximal/distal (%)	77/23	5/95	81/19	52/48
Differentiation, high-moderate/low (%)	67/33	92/8	46/54	86/14
Tumor stage distribution (%)	I:13, II:51, III:36	I:8, II:43, III:49	I:4, II:73, III:15	I:0, II:43, III:57

Non-necrotic tumor areas were macro-dissected and RNA extraction was performed from three 5-*µ*m sections of paraffin-embedded tumor tissue [11], [13] using the High Pure RNA Paraffin Kit (Roche, Castle Hill, Australia) according to the manufacturer’s instructions. RNA concentration was determined using a NanoDrop Spectrophotometer (NanoDrop Technologies, Wilmington, DE) and samples yielding sufficient RNA (200 ng) with 260/280 ratios >1.8 were selected.

### Gene Expression Profiling

Gene expression analyses were performed at the SCIBLU Genomics Centre, Lund University, Sweden. The Illumina Bead-array (HumanWG-6 v4 Expression Beadchip, Illumina) system was used according to the manufacturer’s instructions. Briefly, total RNA was converted to cDNA using biotinylated oligo-dT_18_ and random nonamer primers. Two assay-specific oligonucleotides were designed to interrogate a single contiguous 50 nt sequence on each cDNA. Each of these oligonucleotides consisted of two parts: an upstream-specific oligonucleotide (USO) containing a 3′ gene-specific sequence and a 5′ universal PCR primer sequence (P1), while the downstream-specific oligonucleotide (DSO) contained a 5′ gene-specific sequence and a different 3′ universal PCR primer sequence (P2). Using this approach, a total of 24,526 oligonucleotide pairs (probes) were designed and pooled, which together constituted the whole genome (WG) DASL assay pool (DAP), corresponding to 18,626 unique genes, based on well-annotated content derived from the National Center for Biotechnology Information (NCBI) Reference Sequence Database (Build 36.2, Release 22). The DAP was then annealed to the targeted cDNAs, followed by enzymatic extension and ligation steps, as previously described [19]. Ligated products were PCR-amplified and labeled with a universal Cy3-coupled primer after which single-stranded labeled products were precipitated and hybridized to WG gene expression BeadChips as previously described [20]. BeadChips were then scanned on a BeadArray™ Reader using BeadScan software (v4.2), during which fluorescence intensities were read and images extracted.

### Data Analysis

Expression data were uploaded and processed in the GenomeStudio software (Illumina, San Diego, CA). Data were normalized using background correction, cubic spline method [21] and plate scaling. RefSeq features with a detection *P* value of ≤0.01 in at least 80% of the samples were used, leaving 9,218 features for further analysis. The data were uploaded into MeV v4 [22] where they were log2 transformed and median-centered across assays. Unsupervised clustering was performed on the total set of CRC samples using average linkage clustering with a Pearson correlation as similarity metric. Significance analysis of microarrays (SAM) [23] was used to identify significantly differentially expressed genes with a false-discovery rate (FDR) ***≤***5% [24]. Gene expression data are available in the NCBI Gene Expression Omnibus (GEO) (Accession number GSE36335). Biological pathways were identified using the web-based DAVID software with a FDR ***≤***5% [25].

### Real Time Quantitative Reverse Transcription PCR (qRT-PCR)

qRT-PCR was used to technically validate increased/decreased expression of 5 cancer-related genes with differential expression between the 4 colorectal cancer subsets. The genes were selected to represent major deregulated pathways and expression levels were investigated in 3 samples from each subtype. The *rRNA18S* was used as internal reference gene and normal colon sample as a calibrator. Gene-specific primers and taqman probe sets for each gene were obtained from Applied Biosystems (Applied Biosystems, Foster City, CA). Reverse transcription and PCR was performed using Quantitect reverse transcription kit and probe PCR kit, respectively (Qiagen, Heidelberg, Germany).

### Validation in External Data

The hereditary gene expression signature was validated using the GSE4554 data set and the four largest batches of The Cancer Genome Atlas Network (TCGA) RNAseqv2 [26], [27]. In order to resemble microarray data, the RPKM values of the TCGA were quantile-normalized, an offset of 32 was added, capped at 65,000, and genes were centered. Subsequently the data was adjusted for the batch variable. All data were imported into MeV v4, log2 transformed, genes were median-centered across assays and the 50% least-varying genes were removed. Unsupervised hierarchical clustering was performed as described above. A gene expression centroid was constructed by averaging the expression values of the samples in Lynch syndrome and FCCTX for each gene in order to establish a signature classifier for nearest centroid classification. A sample from the external datasets was assigned to either of the two hereditary subsets based on the maximum Pearson correlation of its centroid expression to the hereditary centroid values.

### Statistical Analysis

Clinical association (tumor location, differentiation, age at onset and stage) in Lynch syndrome and FCCTX were determined using the Fisher’s exact test (with significance set for *P*<0.05). The statistical analyses were performed using the statistical software package IBM SPSS Statistics 20 (SPSS, Chicago, IL, USA).

## Results

Unsupervised hierarchical clustering analysis in the whole series (including 39 Lynch syndrome tumors, 37 FCCTX tumors, 21 sporadic MMR proficient tumors and 26 sporadic MMR deficient tumors) identified two major subgroups related to MMR status (Figure S1). The intrinsic MMR signature was strong with 3873 differentially expressed genes, of which 2159 (56%) were up-regulated in the MMR defective tumors and were related to 5 signaling pathways (Table S1).

**Figure 1:**
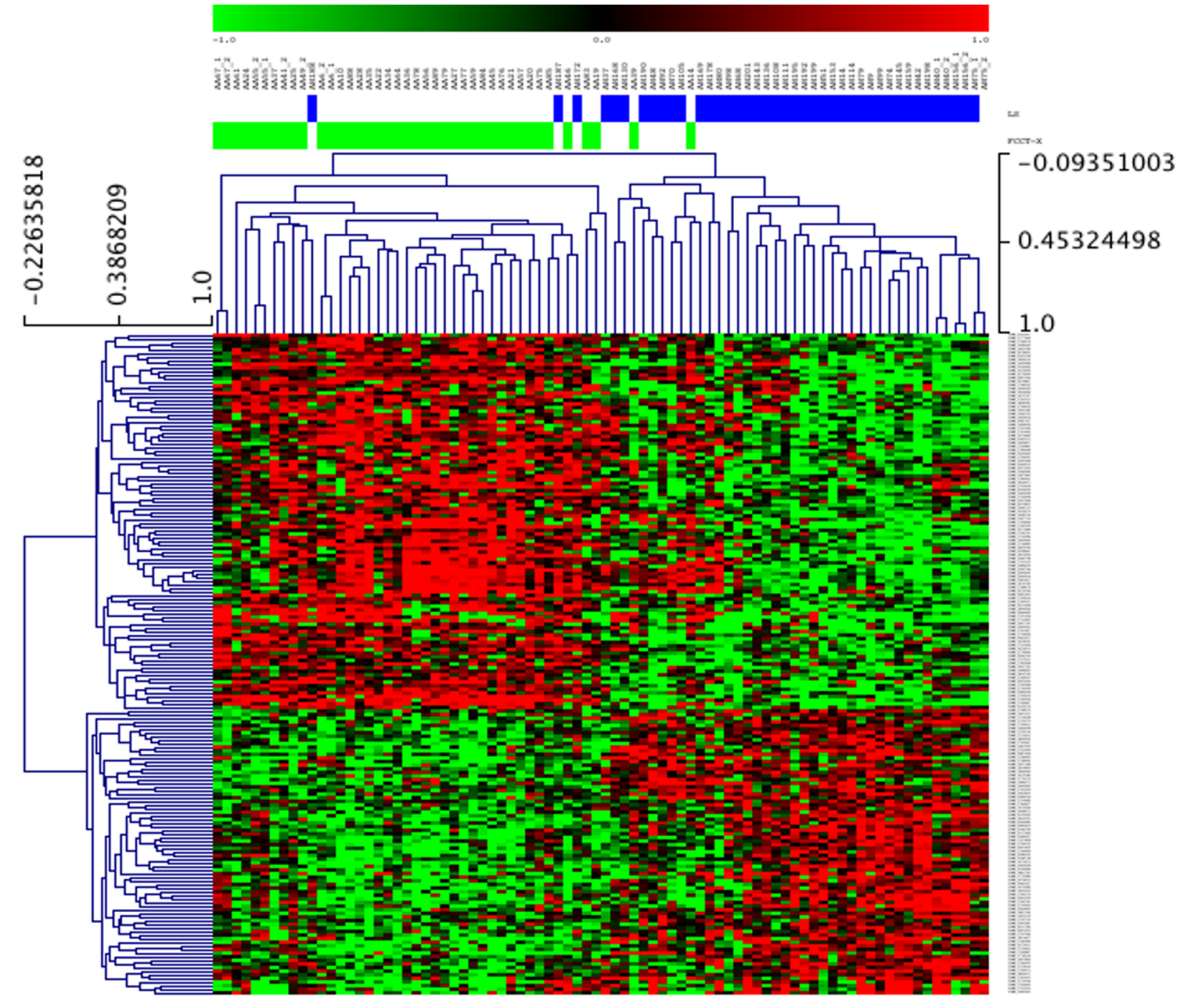
Clustering based on differentially expressed genes between FCCTX and Lynch syndrome tumors, identified by SAM analysis. The figure depicts the top-360 differentially expressed genes. Samples are arranged along the x-axis and show FCCTX tumors (green) and Lynch syndrome tumors (blue).

In the hereditary tumor subset, SAM analysis identified 2188 differentially expressed genes between FCCTX and Lynch syndrome tumors (Figure 1). In FCCTX tumors, the G-protein coupled signaling pathway was up-regulated (FDR = 0.6%), whereas Lynch syndrome tumors showed up-regulation of 2 pathways related to cell cycle and mitosis (FDR = 1.4%) and oxidative phosphorylation (FDR = 3.5%) (Table 2).

**Table 2:** Up-regulated signaling pathways in FCCTX and Lynch syndrome tumors.

Tumor	Pathway	P-value	FDR (%)	Genes
**FCCTX**	Signaling by G-protein coupled receptor	5×10^−4^	0.589	*GNAS, F2R, F2RL2, EDN1, EDNRA, GRM8, GNAZ, GNG11, GNG12, HCRT, PTGER1, P2RY2, RAMP2, MC1R, TUBB3, VIP*
**Tumor**	**Pathway**	**P-value**	**FDR (%)**	**Genes**
**Lynch syndrome**	Cell cycle and mitosis	0.001	1.416	*CDC45, DNA2, E2F2, MIS12, MLF1IP, WEE1, ZWILCH, ANAPC4, ANAPC5, BUB3, CASC5, CDC20, CENPM, CEP135, CCNB2, CCNE1, CCNH, CDK2, CDKN1A, DHFR, FEN1, HSP90AA1, HSP90AA2, KIF2C, KNTC1, MCM4, NUP37, PCNT, PCM1, PTTG1, PTTG2, PLK4, POLE, POLD3, PSMD2, PSMD5, PSME1, PSME2, PSMA4, PSMA5, PSMA6, PSMB2, PSMB4, PSMB6, PPP1CC, PPP2CB, PPP2R5B, RFC5, RPA1, RRM2, STAG1, TYMS, LOC442308, TUBB, TUBBP1, TUBBP2*
	Oxidative phosphorylation	0.003	3.508	*ATP5L, ATP5A1, ATP5B, ATP5D, ATP6V1B2, ATP6V1A, NDUFA7, NDUFA8, NDUFA9, NDUFB2, NDUFAB1, NDUFS2, NDUFV2, TCIRG1, COX7B, COX7C, COX6A1, COX5A, PPA2, LOC100130320, SDHD, SDHA, UQCR10, UQCRFS1, UQCRFSL1, UQCRQ*

The gene expression profiles of FCCTX tumors were closely related to those in sporadic MMR proficient colorectal cancers with genes involved in peptidyl-amino acid modification, enzyme linked receptor protein signaling and growth regulation. Likewise, Lynch syndrome and sporadic MMR defective tumors shared genes involved in cell cycle progression and immune response. Comparison between FCCTX and sporadic MMR proficient tumors revealed 4 differentially expressed genes, whereas comparison between FCCTX and sporadic MMR deficient tumors identified 3906 differentially expressed genes. Lynch syndrome tumors differed from sporadic MMR stable tumors by 415 differentially expressed genes and from sporadic MMR deficient tumors by 91 genes. Among sporadic tumors, 1384 genes differed MMR proficient and deficient tumors.

**Figure 2:**
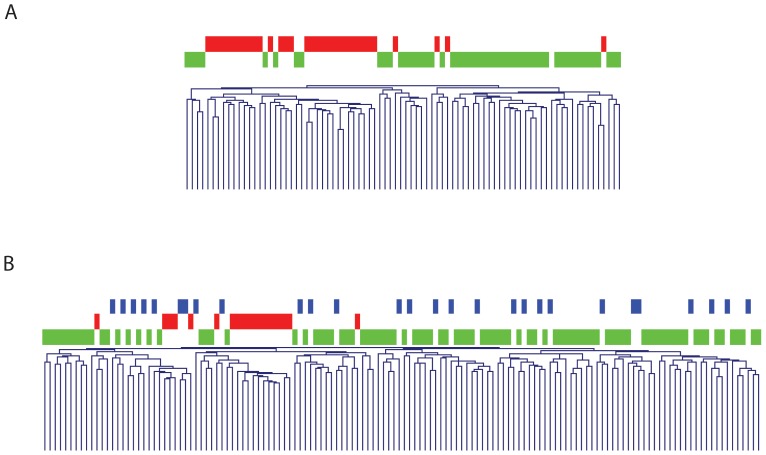
Unsupervised hierarchical clustering based on our 2188-gene signature applied to external data sets. a) Clustering based on GSE4554 and b) Clustering based on the four largest batches of the TCGA RNAseqv2 data sets. MMR proficient tumors (green), microsatellite-low tumors (blue) and MMR deficient tumors (red) along the x-axis.

The genetic profiles were validated in independent datasets, i.e. the GSE4554 (Affymetrix microarray) and the TCGA (RNA sequencing) [26], [27]. Both datasets mainly comprise sporadic colorectal cases, including 51 MMR stable tumors and 33 MMR defective tumors in the GSE4554 data set and 91 MMR stable, 19 MMR defective and 28 MMR-low stable tumors in the TCGA data set. Unsupervised hierarchical clustering based on our 2188 gene signature identified by us, resulted in two major groups related to MMR status. The hereditary signature correctly classified 82% and 80% of the MMR stable tumors as FCCTX and 100% and 94% of the MMR defective tumors as Lynch syndrome, respectively (Figure 2).

**Figure 3:**
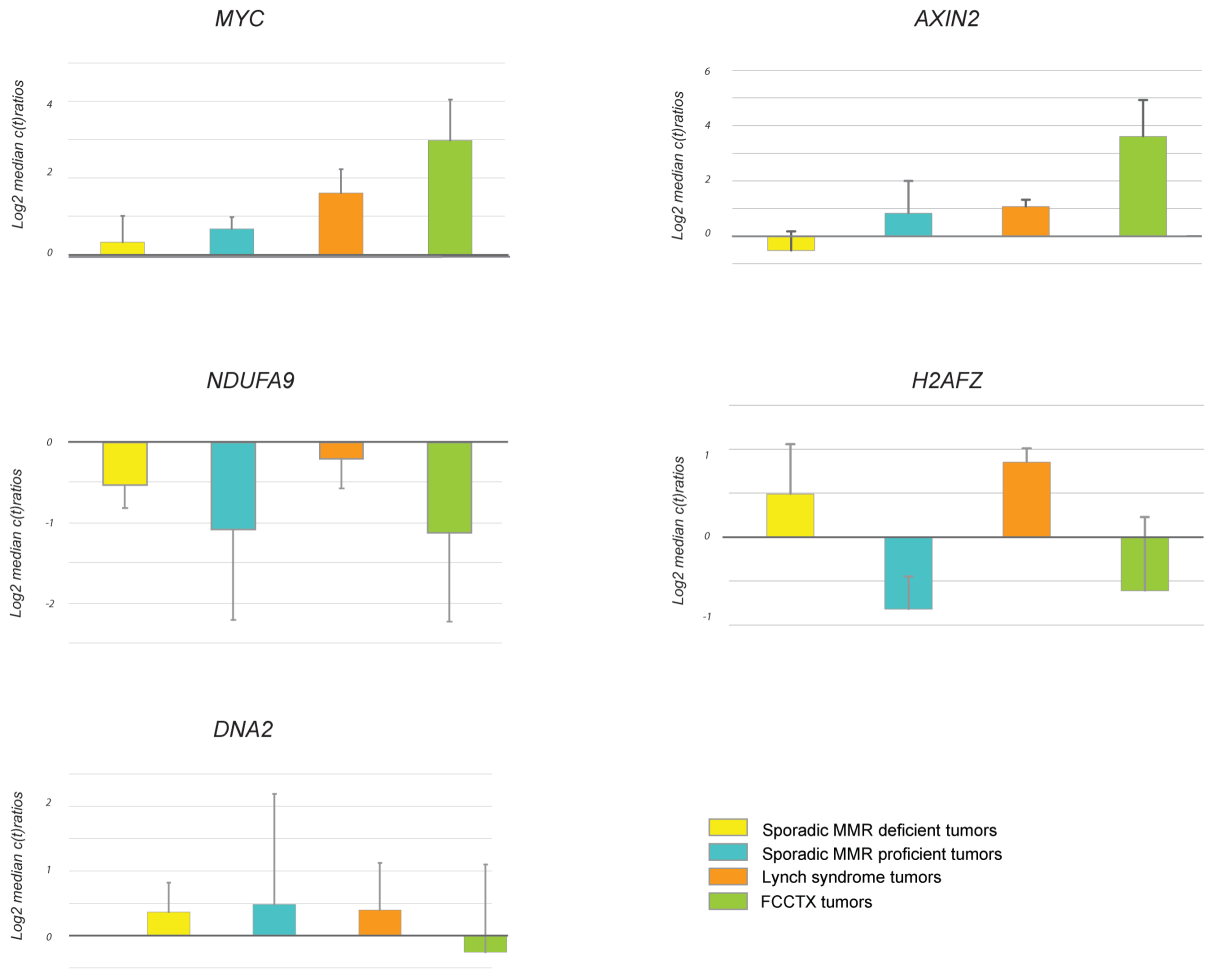
qRT-PCR analysis of 5 target genes in the four different colorectal cancer subsets. Differential expression of *MYC, NDUFA9*, *H2AFZ*, *AXIN2*, and *DNA2* was done for 12 representative samples (3 from each group) and qRT-PCR ratios were normalized to rRNA18S and median centered.

qRT-PCR-based technical validation was performed using 5 cancer-related genes representing deregulated pathways observed in MMR stable and deficient tumors, respectively (Figure 3). The results confirmed increased expression of *MYC* and *AXIN2* in FCCTX tumors, decreased expression of *NDUFA9* in FCCTX tumors and in sporadic MMR proficient tumors and increased expression of *H2AFZ* in MMR deficient tumors. No major differences were observed for *DNA2*.

## Discussion

Within the HNPCC subset of hereditary colorectal cancer, we demonstrated distinctively different gene expression signatures in FCCTX and Lynch syndrome tumors, which differ by 2188 genes. Based on these gene expression differences, our data suggest that MMR status strongly influences genetic signatures, also within phenotypically similar families, which is in agreement to previous studies on sporadic colorectal cancer [16]–[18], [27], [36]. The FCCTX and Lynch syndrome profiles differed in 3 major cancer-related pathways, primarily related to cell cycle progression andmitosis, oxidative phosphorylation and G protein coupled receptor signaling, which have been correlated to colorectal carcinogenesis [18], [28], [29], [36].

The FCCTX tumors showed up-regulation of 1059 genes, several (n = 16) of which were related to the G-protein coupled receptor pathway (Table 2). A candidate target herein includes the *GNAS* gene, which encodes for the G_α_-subunit, and is located in the 20q13.32 region that has been found to be gained in FCCTX tumors [9], [12], [13]. Activating mutations in *GNAS* have been described in CRC and are suggested to promote tumorigenesis through activation of the Wnt and ERK1/2 MAPK signaling pathways [28], [29]. The potential tumorigenic mechanism is unknown and the hot-spot mutation *GNAS* c.601G>T has not been observed in FCCTX tumors [12]. Also other candidate genes involved in proliferation and migration, e.g. *CDH26, SRC* and *ASIP* are located in 20q [30], [31]. The FCCTX tumors also showed up-regulation of *PTGER1,* which encodes for the EP1 receptor that may promote proliferation and colorectal tumor development through altered function of prostaglandin E2 (PGE_2_) [32]–[34].

Lynch syndrome tumors showed up-regulation of 1129 genes, e.g. genes involved in G_1_/S transition (*CCNE*, *CCNH*, *E2F2* and *CDK2*), DNA replication (*CDC45*, *RPA1*, *RFC5*, *MCM4* and *POLD3*) and chromosomal organization and mitosis (*CCNB2*, *MLF1IP*, *CASC5*, *KIF2C* and *PLK4*), which is in line with previous findings (Table 2) [16], [18], [35], [36]. Up-regulation of e.g. *WEE1*, *CDKN1A* and *FANCD2* were also observed in our study and have also been reported by other investigators, which may suggest involvement of the cell cycle checkpoint machinery [37], [38]. Overexpression of checkpoint proteins have previously been linked to Lynch syndrome and abrogation of the checkpoint machinery has been shown to sensitize MMR deficient tumor cells towards chemotherapy [39], [40]. Also genes involved in the oxidative phosphorylation pathway, e.g. the ATP synthase subunit genes and complex I and III subunit genes were among those up-regulated in Lynch syndrome tumors. Down-regulation of ATP synthase subnit genes and complex I, III and V genes have been correlated to metastastic colorectal cancer and may explain the more aggressive tumor development and poor prognosis observed in MMR proficient tumors [11], [18], [41].

In colorectal cancer, MMR status is a major discriminator related to distinctively different gene expression profiles, which is supported by the 3873 differentially expressed genes in our series of MMR deficient and proficient tumors [15], [16], [27]. The importance of MMR status was observed also when we applied the 2188-gene signature that discriminated between Lynch syndrome and FCCTX to two independent data sets mainly containing sporadic tumors. The signature correctly defined 80–82% of the MMR proficient and 94–100% of MMR deficient tumors (Figure 2). Several heat shock proteins and immune response genes (Table S1) were up-regulated in the MMR defective tumors, which supports involvement of immune-response mechanisms, irrespective of whether the tumor was caused by germline or somatic MMR gene inactivation [16]–[18], [42], [43]. In sporadic as well as hereditary MMR proficient tumors several genes in the *Wnt* signaling were up-regulated, which fits well with its central role in colorectal tumorigenesis (Table S1) [44], [45].

In conclusion, hereditary colorectal cancers within the HNPCC subset show distict genetic profiles with 2188 differentially expressed genes between Lynch syndrome and FCCTX. These data pinpoint genes and pathways relevant to further pursue for refined diagnostics and therapeutics in hereditary colorectal cancer.

## Supporting Information

Figure S1 **Unsupervised hierarchical clustering of the entire dataset.** The dendogram shows the spontaneous clustering of 123 colorectal cancers into two major clusters related to MMR status. MMR proficient tumors (green), including FCCTX tumors and sporadic MMR proficient tumors and MMR deficient tumors (blue), including Lynch syndrome tumors and sporadic MMR deficient tumors.
(TIF)

Table S1 **Signaling pathways up-regulated in MMR proficient and MMR deficient tumors.**
(XLS)
