# Supplementary figures and images for: Distinct Gene Expression Signatures in Lynch Syndrome and Familial Colorectal Cancer Type X
Source: PLoS One. 2013 Aug 12;8(8):e71755. doi: 10.1371/journal.pone.0071755 (PMC3741139; doi:10.1371/journal.pone.0071755)

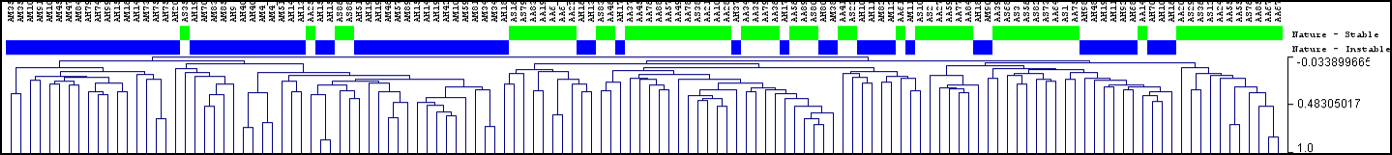

Supplement: Figure S1 — Unsupervised hierarchical clustering of the entire dataset. The dendogram shows the spontaneous clustering of 123 colorectal cancers into two major clusters related to MMR status. MMR proficient tumors (green), including FCCTX tumors and sporadic MMR proficient tumors and MMR deficient tumors (blue), including Lynch syndrome tumors and sporadic MMR deficient tumors. (TIF) [file pone.0071755.s001.tif]
